# Atom‐in‐Molecule Analysis, Density Function Theory Research, Spectroscopy, Charge Transfer, and (Static, Dynamic) Nonlinear Optical Effects of (Z)4,4^′^‐Bis[‐3‐N‐Ethyl‐2‐N’‐(Phenylimino) Thiazolidin‐4‐One] Methane

**DOI:** 10.1002/open.70205

**Published:** 2026-05-21

**Authors:** Zohra Douaa Benyahlou, Fayssal Triki Baara, Salem Yahiaoui, Merzouk Saidj, Keltoum Dermeche, Mohammed Harir, Youcef Megrouss, Ahmed Djafri, Abdelkader Chouaih, Hamdi Bendif, Walid Elfalleh, Tarek H. Taha, Anis Ahmad Chaudhary, Stefania Garzoli

**Affiliations:** ^1^ Process Engineering Department Laboratory of Technology and Solid Properties Faculty of Sciences and Technology Abdelhamid Ibn Badis University Mostaganem Algeria; ^2^ Laboratory of Environment and Sustainable Development Faculty of Nature and Life Sciences University of Ahmed Zabana Relizane Algeria; ^3^ Higher Normal School of Mostaganem Mostaganem Algeria; ^4^ Faculty of Nature and Life Sciences University of Sciences and Technology of Oran Oran Algeria; ^5^ Chemistry Department Faculty of Exact Sciences and Informatic Hassiba Benbouali University Chlef Algeria; ^6^ Organic Synthesis Division Centre de Recherche Scientifique et Technique en Analyses Physico‐Chimiques (CRAPC) Tipaza Algeria; ^7^ Department of Biology College of Science Imam Mohammad Ibn Saud Islamic University (IMSIU) Riyadh Saudi Arabia; ^8^ Department of Chemistry and Technologies of Drug Sapienza University Rome Italy

**Keywords:** Becke–Yang‐part function, charge transfer, energy gap, nonlinear optical, X‐ray diffraction

## Abstract

The purpose of this research is to report the outcomes of experimental and theoretical spectroscopic analyses of (Z)4,4^′^‐bis[‐3‐N‐ethyl‐2‐N’‐(phenylimino) thiazolidin‐4‐one] methane (2‐EPTh). Fourier transform infrared (FT‐IR), UV–vis, 1H, and 13C nuclear magnetic resonance techniques characterized the molecule's structure. UV–vis results indicated that the molecule can absorb light in the wavelength range of 200–375 nm. Density functional theory calculations have been performed using Becke–Yang‐part function functional with the basis set 6‐311G (d, p) to support the experimental results. Theoretical and empirical findings are approximately similar. In addition, the molecular electrostatic potential (ESP) of 2‐EPTh was simulated to identify favorable sites for electrophilic and nucleophilic reactions. Moreover, the frontier orbital energy gap was thoroughly studied. Furthermore, atom‐in‐molecules theory examines covalent bonding and intermolecular interactions. The ESP and dipole moment were also determined using experimental X‐ray diffraction data. Ultimately, the study determined that the title compound is a suitable candidate for nonlinear optical applications, as demonstrated by its electric dipole moment (μ), polarizability (α), and first hyperpolarizability (β). More specifically, its second hyperpolarizability response has shown promising results for the dimer of our molecule, in both static and dynamic dependent‐frequency (λ = 532 nm) states, and in the gaseous environment as well as in the presence of solvent.

## Graphical abstract

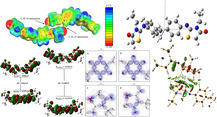

## 1 Introduction

The interest in nonlinear optical (NLO) materials has experienced a substantial surge in recent years [1, 2, 3], primarily due to their considerable potential for utilization in optoelectronic devices [4]. These materials include heterocyclic compounds with pentagonal rings, nitrogen, sulfur, and oxygen atoms. Thiazole derivatives, namely thiazolidinone molecules, belong to a significant class of chemical products [5]. Chemists and physicists have recently shown great interest in the push‐pull effects [6] of thiazolidinone compounds due to their notable linear and NLOs characteristics [7, 8, 9, 10, 11, 12]. The quest for novel NLO materials mostly depends on the theoretical forecasting of accurate electro‐optical characteristics. Computational techniques can compute qualities that have yet to be documented by experimentation. Conventional experimental methods, such as nuclear magnetic resonance (NMR) spectroscopy, infrared (IR) spectroscopy, X‐ray crystallography, and other spectroscopic techniques, do not entirely characterize a molecular structure. Nevertheless, these procedures are employed in conjunction with theoretical methodologies. Furthermore, computational approaches can serve as a crucial instrument to enhance the capacity and effectiveness of the elucidation process.

**FIGURE 1:**
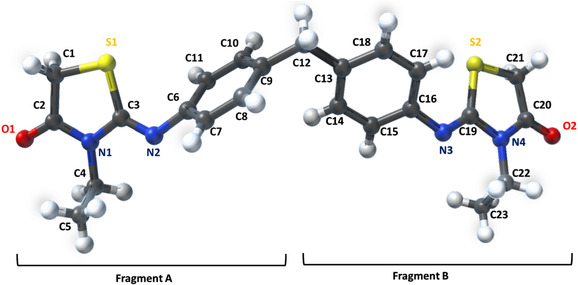
Molecular structure of 2‐EPTh.

The work, which has been recently published [13], centers on the creation and analysis of a novel chemical molecule called (Z)4,4^′^‐bis[‐3‐N‐ethyl‐2‐N’‐(phenylimino) thiazolidin‐4‐one] methane (2‐EPTh) (Figure 1). The molecule's intermolecular interactions were examined using the Hirshfield surface and reduced density gradient. The provided information was utilized in the development of a novel pharmaceutical compound. This study combines experimental methods and molecular modeling to examine the structural characteristics, vibrational frequencies, 1H and 13C NMR chemical shifts, and UV–vis electronic transitions. An experimental electrostatic potential (ESP) map derived from the X‐ray charge‐density model was compared with the density functional theory (DFT)‐based molecular electrostatic potential (MEP) to evaluate the consistency between theoretical and experimental charge distributions. Theoretical calculations are performed to determine the highest occupied molecular orbital (HOMO)‐lowest unoccupied molecular orbital (LUMO) energies of the title chemical. This analysis aims to gain insights into the compound's reactive nature and charge transfer properties.

Our goal is to understand the NLO properties of the crystal formed by our molecule 2‐EPTh. Therefore, it may be more appropriate to study the dimer as it can provide more relevant insights into the effects of various interactions on the properties of the crystalline material. As a result, we conducted a comparative study between the molecule in dimer and monomer states, both statically and dynamically, using a 532 nm frequency. Additionally, we explored the influence of the environment on the NLO response in both gas and in the presence of a polar protic solvent (Ethanol). Various parameters, including dipole moment (μ), polarizability (α), first hyperpolarizability (β), and second hyperpolarizability (γ), were measured to demonstrate the structure's favorable performance in the field of nonlinear optics. A dipole moment experimental investigation was conducted to corroborate the theoretical findings. Our study of atom‐in‐molecule (AIM) theory has allowed us to gain a deeper understanding of the electrical structure of molecules by focusing on localized atomic interactions. The AIM theory proposes that the arrangement of electrons within a molecule can be understood by examining electron density. Furthermore, AIM theory elucidates the characteristics of chemical interactions and the formation of bonds inside molecules.

Theoretical calculations in this study utilized DFT with the three‐parameter hybrid Becke–Yang‐part function (B3LYP) at the 6‐311G (d, p) basis set. The DFT calculation achieved a commendable balance between several factors, including experimental and computational efficiency.

## 2 Material and Methods

### 2.1 Spectral Data Measurements

Nicolet fourier transform infrared (FT‐IR) 6700 spectrometer was used to record the IR spectrum of (Z)4,4^′^‐bis[‐3‐N‐ethyl‐2‐N’‐(phenylimino) thiazolidin‐4‐one] methane in the range of 450–4000 cm^−1^ with KBr pellets as the sample. ^1^H NMR (500 MHz) and ^13^C NMR (125 MHz) spectra of the molecule were obtained using a Bruker AC300 MHz spectrometer in a solid medium at 298 K. An electronic spectrum of 2‐EPTh was also measured using a spectrophotometer Optizen 2120UV with a 190.0nm  ∼ 1100.0 nm wavelength range.

### 2.2 Theoretical Approaches and Computational Details

All DFT calculations for this theoretical investigation were executed utilizing the Gaussian 09 [14] and GaussView 6 programs [15]. The hybrid functional B3LYP with the basis set 6‐311G (d, p) was utilized in every calculation. The compound's molecular structure under investigation was optimized using the X‐ray structure as the initial geometry. The dimer structure used in the calculations was constructed from the crystallographic data obtained from X‐ray diffraction. The dimer was extracted from the crystal packing using the CIF file and subsequently optimized at the B3LYP/6‐311G(d, p) level. Vibrational frequencies were calculated to verify that the optimized geometries align with the compound's global minimum energy structure. Spectroscopic analysis of 1H and 13C NMR chemical shifts and UV–vis transitions has confirmed the structure. To predict the reactive sites for electrophilic or nucleophilic attack on the given compound, the electronic properties, including the energy values of the HOMO and LUMO and MEP, were computed using the same levels of theory. To evaluate the NLO properties of the compound in question, the following parameters were computed: dipole moment (μ), average polarizability (α), first hyperpolarizability (β), and second hyperpolarizability (γ). The frequency‐dependent first and second hyperpolarizabilities (β and γ) were calculated using the coupled‐perturbed Kohn–Sham method implemented in Gaussian at the B3LYP/6‐311G (d, p) level. Dynamic NLO properties were evaluated at an incident wavelength of 532 nm.

## 3 Results and Discussion

### 3.1 Vibrational Frequencies

**FIGURE 2:**
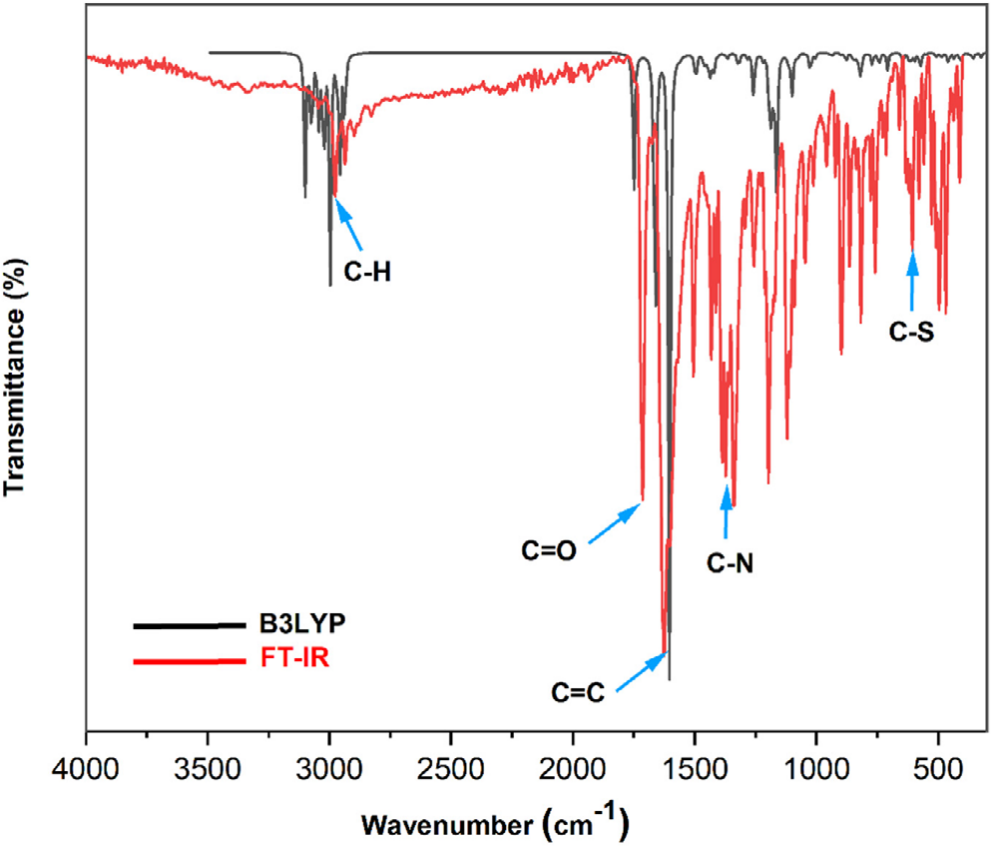
Theoretical and experimental FT‐IR spectra of 2‐EPTh.

Theoretical analysis of IR spectroscopy for the compound 2‐EPTh is based on the DFT calculation using the B3LYP method with a 6‐311G (d, p) basis set. Gaussian program and GaussView were used to perform the calculation. Wavenumber calculations confirmed the optimized geometry's stability, which gave positive values for all the obtained wavenumbers [16]. A frequency‐scaled factor of 0.973 was employed for the vibrational analysis because DFT‐calculated harmonic vibrational frequencies are usually larger than those observed experimentally [17]. Vibrational assignments were made based on calculating the potential energy distribution (PED) using the VEDA 4 program [18]. Table S1 summarizes the results obtained from the calculated frequencies (scaled and unscaled) and the experimental study. Figure 2 shows the superposition of the theoretical and experimental IR spectrum.

**C—H vibrations.** In the aromatic compounds, the peaks due to the C–H stretching vibrations appear in the spectral range 3100–3000 cm^−1^ [19]. This peak is calculated at 3098 cm‐1 and 3071 cm‐1 frequency regions in the present study. As they are also detected in the IR spectrum, respectively at 3099 and 3072 cm^−1^. The C–H in‐plane bending bands of the aromatic molecules are found in the spectral range 1300–1000 cm^−1^ [20]. The H–C–C symmetrical and asymmetrical link in plane bending vibrations, combined with other vibration bands, are experimentally observed at 1341 and 1256 cm‐1 for the two aromatic rings. The corresponding peaks in the theoretical spectrum are calculated at 1310 and 1256 cm^−1^.

**C—C vibrations.** Appearance of C=C stretching vibrations of the aromatic rings typically falls within the spectral range of 1650–1200 cm^−1^ [21, 22]. From the experimental spectra of FT‐IR, the C=C stretching vibrations are found at 1627 cm^−1^ with a large band, comparable to theoretical results at 1604 cm^−1^ for the two aromatic rings of 2‐EPTh. The observed band in IR at 1198 cm^−1^ belongs to the symmetric C–C stretching vibration and, on the other hand, to the asymmetric C–C stretching vibration. Symmetric and asymmetric C–C–C bending modes of aromatic rings are observed at 1010 cm^−1^, and the respective calculated band is assigned at 1005 cm^−1^. We observed the C–C stretching vibration for the thiazol ring at 1046 cm‐1, comparable to theoretical data at 1024 and 1025 cm^−1^. The computed vibration band at 508 cm^−1^ is assigned to C–C–C–C asymmetric out‐of‐plane bending, comparable to experimental data at 526 cm^−1^.

**C=O vibration.** Carbonyl group bond stretching vibration generally appears in the 1850–1550 cm^−1^ [23] spectral range. The π–π bond between carbon and oxygen forms a double bond between them. The distribution of electrons in this bond is unequal because these atoms have various electronegativities. The unpaired electrons on the oxygen are responsible for the polar character of the carbonyl group. For our compound, the C=O stretching vibrations are observed at 1721 cm^−1^ in the FT‐IR spectra. This stretching peak is also computed at 1750 and 1749 cm^−1^.

**C–N vibrations.** It isn’t easy to recognize C–N vibrations in an FT‐IR spectrum, because in this region, 1382–1266 cm^−1^, it is possible to have a mixture of several bands [24]. In the present study, the calculated stretching mode found at 1366 cm^−1^ is assigned to the C–N stretching band for the two rings of thiazolidinone. This value is in agreement with the observed FT‐IR values at 1366 cm^−1^. An asymmetric stretching of the C–N vibration appears in the experimental spectra IR at 1046 cm^−1^, corresponding to the theoretical values at 1024 and 1025 cm^−1^. A bending vibration of C–C–N is shown at 759 cm^−1^ in the spectrum of the headed compound, coinciding with 745 cm^−1^ of the theoretical calculations [25].

**C–S vibrations.** It is difficult to identify the C–S stretching vibration in various compounds, as it has a variable intensity and can be found in the broad region of 1035–245 cm^−1^ in both aliphatic and aromatic molecules [26]. The theoretical wavenumber was calculated at 607 cm^−1^, corresponding to C–S stretching vibrations with a PED of 81%.

**CH**_**2**_ **, CH**_**3**_ **group vibrations.** In the literature, the different modes of C–H vibrations for the CH_2_ and CH_3_ groups have multiple absorption bands between 2900–3000 cm^−1^ [27]. An asymmetric and a symmetric C–H stretching mode of CH_2_ are found at 2976 cm^−1^ in FT‐IR spectra, comparable to the theoretical value at 2967 cm^−1^. Even for the stretching vibration of the CH_3_ group, the observed value (2956 cm^−1^) corresponds to the theoretical one (2957 cm^−1^).

Results show that the experimental and theoretical results are highly similar and that these assignments agree with the literature [20, 28].

### 3.2 UV–Visible

Ultraviolet–visible spectroscopy is about the interactions between electromagnetic radiation and material in the ultraviolet–visible region. Specifically, it is related to the excitation of atoms’ outermost electrons, which are implicated in forming molecules and are therefore often called “electron spectroscopy” [29, 30]. Using the TD‐DFT method for the 2‐EPTh compound, electronic transitions were investigated from the optimized structure obtained by B3LYP/6‐311G(d, p) level. Results indicating vertical excitation energies, oscillator strength (f), transition wavelength, and contributions are grouped in Table 1.

**TABLE 1:** Wavelength, energy, and MO contributions (≥ 10%) obtained from the study of the UV–Visible spectrum of the title molecule.

No	**Energy, cm** ^**−1**^	Wavelength λ, nm		MO contributions
		Theoretical	Experimental	
1	35 058.74352	285	315	HOMO→LUMO (75%) H‐1→L + 1 (19%)
2	35 612.85024	281	/	HOMO‐>L + 1 (67%) H‐1→LUMO (21%)
3	38 069.632	263	244	HOMO→L + 2 (48%) H‐1→L + 1 (19%)

**FIGURE 3:**
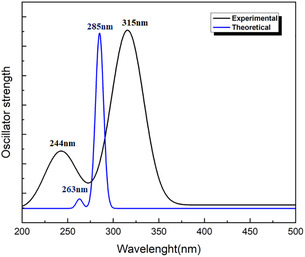
Experimental and calculated UV–visible absorption spectra of 2‐EPTh in the gas phase.

Additionally, Figure 3 depicts the experimental and theoretical UV–visible absorption spectra. The electronic spectra of the compound of interest exhibit absorption bands within the 200–375 nm range. The experimental spectrum exhibited two peaks at 244 and 315 nm in the ultraviolet region. The corresponding calculated absorption peaks have been found first, at λ = 263 nm, which corresponds to the electronic excitation contributions from HOMO and HOMO‐1 to LUMO + 2 (48%) and LUMO + 1 (19%), respectively. The second peak was calculated at λ = 285 nm. This value represents the maximum absorbance for the first excited state with an excitation energy of 4.3467 eV. In this state, the electronic transitions are HOMO→LUMO (75%) and HOMO‐1→LUMO + 1 (19%). The simulated transition at 281 nm is a combination of HOMO→LUMO + 1 (67%) and HOMO‐1→LUMO (21%).

The assignment of the electronic transitions was established based on the analysis of the frontier molecular orbitals. The HOMO orbital is mainly delocalized over the π‐conjugated framework of the molecule, while the LUMO orbital is distributed over the corresponding antibonding π* orbitals, indicating that the dominant HOMO→LUMO excitation corresponds to a π→π* transition. In contrast, the HOMO‐1 orbital exhibits electron density contributions associated with lone pairs located on heteroatoms such as nitrogen and sulfur, suggesting possible n→π* transitions. Consequently, the absorption bands observed around 285 and 315 nm can be attributed mainly to π→π* transitions, whereas the bands around 263 and 244 nm involve contributions from n→π* excitations [31, 32]. The total density of states (DOS) curve for 2‐EPTh obtained using the GaussSum 3.0 program is shown in Figure S1 [24].

The calculated HOMO–LUMO energy gap (Eg = 5.012 eV) indicates a relatively large electronic gap. However, it should be noted that the HOMO–LUMO gap obtained from DFT calculations does not correspond directly to the optical bandgap. The transparency of the compound is mainly supported by the experimental UV–vis spectrum, which shows absorption bands only in the ultraviolet region (200–375 nm), with no absorption in the visible range (400–700 nm). This suggests that the compound is transparent and appears colorless.

### 3.3 NMR Spectral Analysis

NMR spectroscopy is one of the most powerful tools for structure determination of both organic and inorganic species. The structure of 2‐EPTh was confirmed by theoretical and experimental ^1^H NMR and ^13^C NMR spectral study. The chemical shift has a great importance in NMR spectroscopy [33]. It is located in relation to a reference frequency which is tetramethylsilane (TMS), hence its NMR spectrum was drawn up after the calculation performed on the optimization of its geometry using the same level B3LYP/6‐311G(d, p) with the gage‐including atomic orbital (GIAO), continuous set of gage transformations (CSGT) and individual gages for atoms in molecule (IGAIM) approaches of the theoretical calculation of the compound 2‐EPTh [34, 35]. The experimental and theoretical chemical shifts are reported in Table 2.

**TABLE 2:** Experimental and calculated ^1^H and ^13^C NMR chemical shifts data (ppm).

Atoms	B3LYP 6–311 G, d, p			Experimental
	GIAO IGAIM CSGT			
^**13**^ **C**				
C20	177.070	170.501	170.473	171.544
C2	176.974	170.491	170.463	171.544
C19	162.137	157.439	157.410	153.811
C3	162.273	157.741	157.712	153.811
C16	156.507	150.945	150.928	146.022
C6	156.572	151.082	151.066	146.022
C13	148.918	144.489	144.470	137.387
C9	148.696	144.243	144.224	137.387
C14	139.346	135.515	135.506	129.742
C10	138.848	135.281	135.272	129.742
C8	138.416	134.637	134.630	129.742
C18	137.313	133.695	133.688	129.742
C7	129.743	126.034	126.030	121.035
C17	125.510	121.408	121.401	121.035
C15	131.409	127.787	127.781	109.987
C11	127.090	123.250	123.243	109.987
C4	45.770	45.018	45.005	40.824
C22	45.737	45.045	45.031	40.824
C12	49.572	47.610	47.602	38.289
C21	42.250	38.310	38.306	32.764
C1	42.321	38.402	38.397	32.764
C23	16.265	16.627	16.622	12.521
C5	16.256	16.610	16.606	12.521
^**1**^ **H**	—	—	—	—
H8	8.185	6.983	6.984	6.892
H10	7.837	6.897	6.894	6.892
H18	8.060	6.868	6.869	6.892
H7	7.725	6.464	6.465	6.892
H17	7.548	6.336	6.335	6.892
H11	7.508	6.404	6.403	6.892
H15	7.665	6.474	6.474	6.892
H14	7.890	6.919	6.916	6.892
H12A	4.539	3.551	3.549	5.289
H12B	4.557	3.572	3.571	5.289
H4B	4.511	3.682	3.677	3.783
H4A	4.415	3.748	3.743	3.783
H22B	4.489	3.669	3.664	3.783
H22A	4.402	3.743	3.738	3.783
H21B	4.173	2.721	2.723	2.038
H1B	4.162	2.719	2.721	2.038
H21A	4.148	2.717	2.719	2.038
H1A	4.148	2.725	2.727	2.038
H5B	2.256	1.892	1.889	1.269
H23C	1.777	1.485	1.484	1.269
H5C	1.815	1.501	1.499	1.269
H23A	1.526	1.047	1.046	1.269
H5A	1.565	1.069	1.068	1.269
H23B	2.179	1.846	1.843	1.269

From the obtained results, it can be seen that the chemical displacement of the atoms is almost identical to the two fragments (A and B) of the studied molecule, which is due to the presence of a symmetry at the C12 atom. It is known that the ^13^C NMR chemical shifts for the aromatic C atom give signals in overlapping areas of the spectrum with values from 100 to 170 ppm. In this work, the chemical shift values of aromatic carbon were observed in the range 109.987–146.022 ppm, while the theoretical calculation values are 127.090–156.507 ppm, 123.250–150.945 ppm, and 123.243–150.928 ppm for GIAO, IGAIM, and CSGT, respectively.

The higher electronegativity of nitrogen (N) and oxygen (O) atoms can polarize the electron distribution in their bonds with adjacent carbon atoms, leading to reduced chemical shift values. For example, carbon atom C19, surrounded by N4 and N3 atoms, exhibits a high experimental chemical shift value of 153.811 ppm. Theoretical calculations using GIAO, IGAIM, and CSGT methods predict values of 162.137, 157.439, and 157.410 ppm, respectively. This suggests that the presence of neighboring N atoms is indeed influencing the carbon's chemical shift. The largest value of chemical shift in this molecule was observed at 171.544 ppm for the carbon C20, which is bound to the O2 and N4 atoms, while the calculation of GIAO, IGAIM, and CSGT has been given at 177.070, 170.501, and 170.473 ppm, respectively. This shows that oxygen has a great influence on the chemical shift. These results are almost identical to those of the second fragment.

In the ^1^H NMR spectrum of the compound, the characteristic proton resonances of the molecule are observed. The ^1^H NMR signal corresponding to the CH_2_ protons of the benzyl group (H12A and H12B) appears experimentally at 5.289 ppm, while the calculated values range between 3.55 and 4.56 ppm depending on the computational method. This deviation may be attributed to environmental effects such as solvent interactions and anisotropic shielding from nearby aromatic rings, which are not fully captured in the gas‐phase calculations. The peaks of the aromatic protons of the phenyl group were identified experimentally at 6.892 ppm and computed in the region of 8.185–7.890 ppm, 6.983–6.919 ppm, and 6.984–6.916 ppm for GIAO, IGAIM, and CSGT, respectively. The benzyl group's methyl protons (CH_3_) chemical shift was determined to be in the 1.269 ppm range. Finally, we conclude that in solid‐state NMR, chemical shift values depend strongly on the crystal structure of the sample and its symmetry. The peak splitting diagram in the complex spectrum was found to be consistent with the structure [36, 37]. It is also added that IGAIM and CSGT approaches are extensions and improvements of the GIAO and can provide more accurate results for complex molecular and solid systems. That's why the results obtained by IGAIM and CSGT may be closer to the experimental data than those obtained by the GIAO approximation, especially for chemical systems that exhibit complex dipolar interactions or complicated molecular environments.

### 3.4 HOMO‐LUMO Analysis

Frontier molecular orbitals, also known as FMO, are named as such because the HOMO corresponds to the HOMOs, indicating its ability to donate an electron [38]. On the other hand, the LUMO corresponds to the LUMOs, indicating its ability to accept an electron [39]. The difference in energy between the HOMO and LUMO determines chemical reactivity, optical polarizability, and chemical hardness‐softness of the studied systems. [40, 41].

**FIGURE 4:**
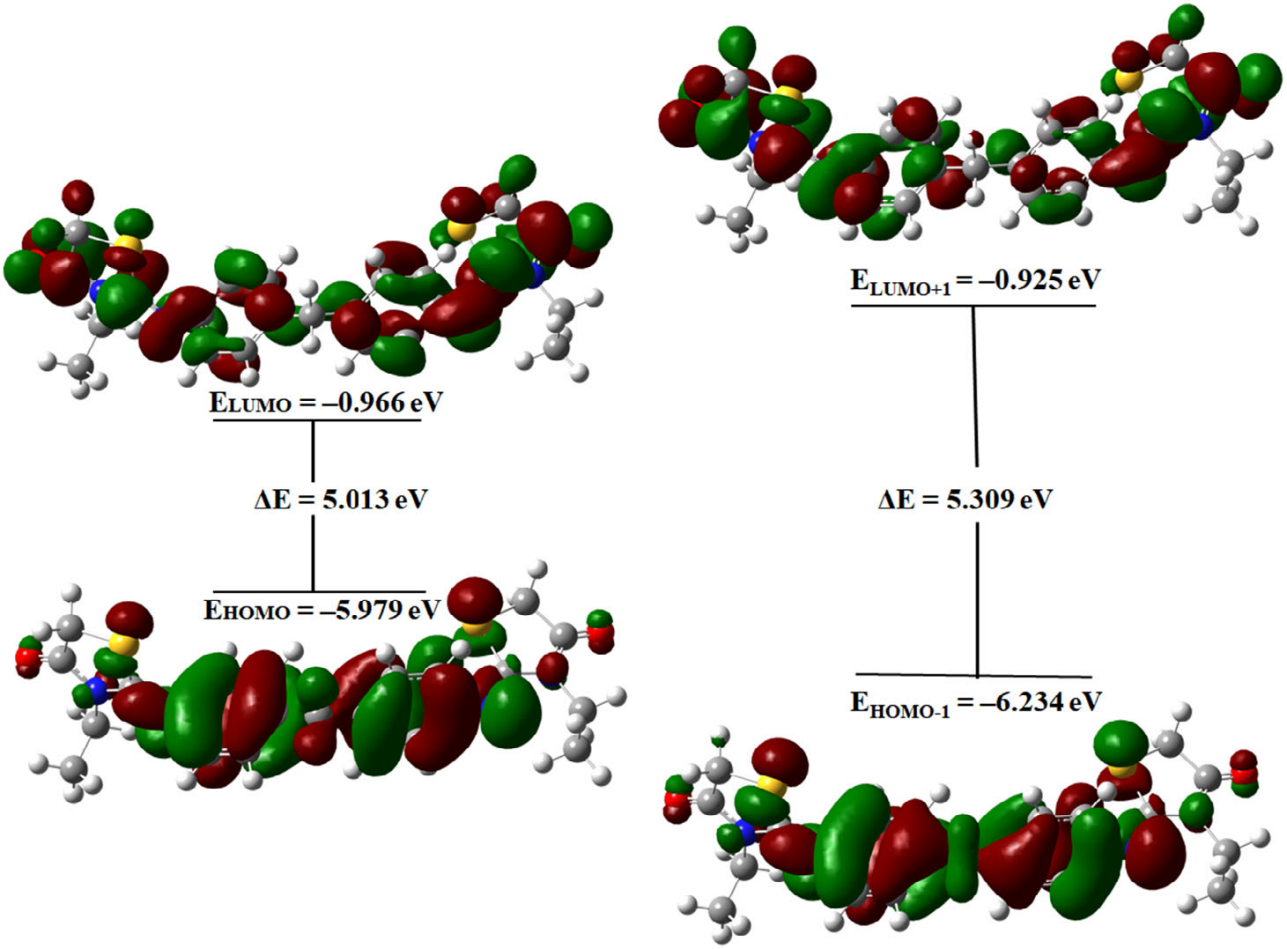
Molecular orbital surfaces for 2‐EPTh.

The current investigation involves calculating the energies of the HOMO‐LUMO using the B3LYP method and the 6‐311G (d, p) basis set. According to Figure 4, the HOMO is mainly localized over the π‐conjugated aromatic rings of the molecule, indicating that this orbital has predominantly π character. When electrons migrate to the thiazolidinone rings, the move from HOMO to LUMO happens. Large aromatic systems, in particular, exhibit increased mobility of π electrons when small HOMO‐LUMO gaps exist. This is due to the ease with which electrons can transition to higher energy levels closely matched in energy. The HOMO‐LUMO gap for 2‐EPTh has a value of 5.01288 eV. This result suggests that the HOMO exhibits nucleophilic character, while the LUMO shows electrophilic character, facilitating intramolecular charge transfer within the conjugated framework. The parameters μ and β exhibit an increasing trend as the HOMO‐LUMO energy gap decreases. A smaller HOMO–LUMO energy gap generally indicates higher electronic polarizability and enhanced charge–transfer capability within the molecular system.

The HOMO−1 orbital shows electron density contributions associated with heteroatoms such as nitrogen and sulfur, indicating the presence of nonbonding (n) orbitals. In contrast, the LUMO and LUMO + 1 orbitals are primarily localized on the molecule's conjugated π framework, corresponding to antibonding π* orbitals. This orbital distribution suggests that the dominant electronic transitions mainly involve π→π* excitations within the conjugated system, with possible n→π* contributions from lone‐pair orbitals of the heteroatoms.

### 3.5 Global Chemical Reactivity Descriptors

To understand the chemical reactivity and stability of 2‐EPTh, electronic properties are calculated as descriptors of the global chemical reactivity: electronegativity χ, chemical potential P, global softness S, electrophilicity index ω, and global hardness η. These parameters are calculated from the ionization potential I_p_ and electron affinity E_A_, which are related to the value of HOMO and LUMO compound studied is chemically stable due to its high gap value (5.012 eV). Global reactivity parameters of this study were calculated using the B3LYP function by 6‐311G (d, p) basis set with the following equations [42]:+

$$I_{p}=E_{\mathrm{HOMO}}\;\;\;;\;\;\;E_{A}=E_{\mathrm{LUMO}}\;\;\;:\;\;\;\chi =\frac{1}{2}(E_{\mathrm{LUMO}}+E_{\mathrm{HOMO}})\;\;\;:P=\frac{1}{2}(E_{\mathrm{LUMO}}+E_{\mathrm{HOMO}})\;\;\;;\;\;\;\eta =\frac{1}{2}(E_{\mathrm{LUMO}}‐E_{\mathrm{HOMO}})\;\;\;;S=\frac{1}{\eta }\;\;\;;\;\;\;\omega =\frac{P^{2}}{2\eta }\;\;\;;\;\;\;\Gamma =E_{\mathrm{LUMO}}‐2E_{\mathrm{HOMO}}+E_{\mathrm{HOMO}‐1}$$

Results of the calculation are collected in Table 3. The ionization potential of this molecule is estimated with a high value (5.98 eV), which indicates a high stability and chemical inertness. In addition, a positive value (0.97 eV) of the electron affinity showed that the compound has the ability to form anions. A negative value of chemical potential (−3.48 eV) was found, which indicates that it is more difficult for the compound to lose an electron but easier to gain one. This result can be associated with the molecular electronegativity (3.48 eV). In addition, the result of the calculation provided the values of electrophilicity, chemical hardness, and chemical softness: 2.41, 2.51, 0.40 eV, respectively. The molecule's stability is further reinforced by its chemical potential value, which remains constant, and a hyper‐hardness value (4.76 eV) that is positive. They are a useful concept to understand the behavior of chemical systems. It was found that the 2‐EPTh is a hard molecule that has a large energy gap.

**TABLE 3:** Calculated global reactivity descriptors (eV) for 2‐EPTh.

Parameters	B3LYP/6‐311G, d, p
E_HOMO_ (eV)	−5.98
E_LUMO_ (eV)	−0.97
E_HOMO_ − E_LUMO_ (eV)	5.01
I_p_ (eV)	5.98
E_A_ (eV)	0.97
χ (eV)	3.48
P (eV)	−3.48
η (eV)	2.51
S (eV)	0.40
ω (eV)	2.41
Γ (eV)	4.76

### 3.6 MEP

MEP is associated with the electron density. The charge distribution generally presents the MEP in the space around the molecule. The MEP is a very useful descriptor for determining the sites of electrophilic reactions, indicated by the negative (red and yellow) regions of the MEP, and nucleophilic reactions are represented by the positive (blue) regions. It can also determine hydrogen bonding interactions [43]. The MEP clearly shows the relationship between the physicochemical properties of the molecule and its structure [44, 45 **]** **.** Among them, the molecular dipole moment can be calculated.

**FIGURE 5:**
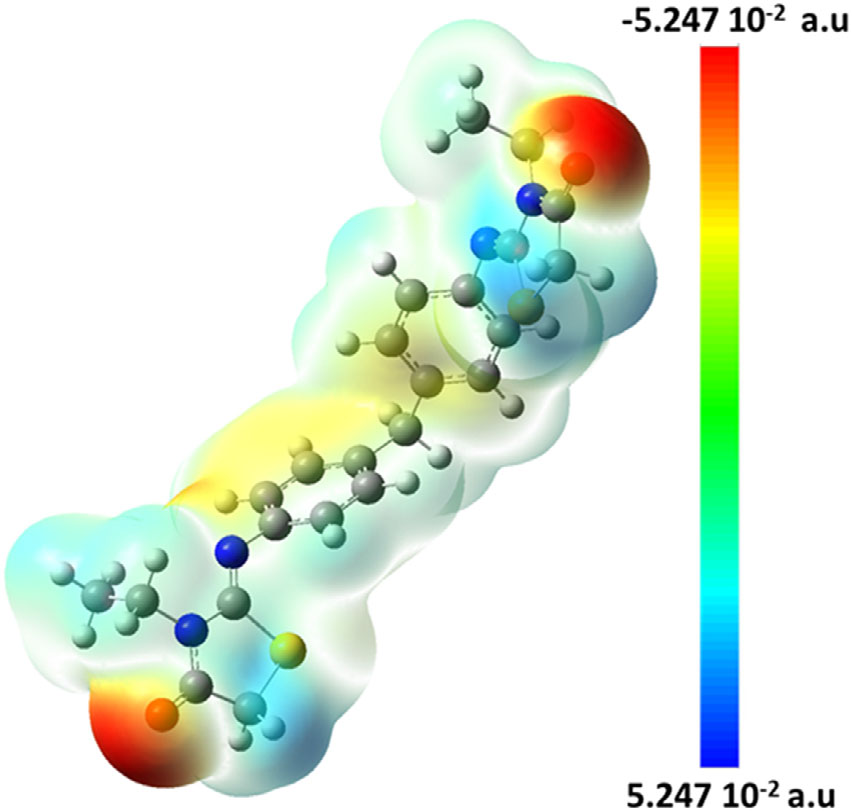
2‐EPTh MEP.

The ESP surface was plotted for the title compound in the B3LYP/6‐311G(d, p) level; the result is shown in Figure 5. The color coding from −5.247.10^−2^ to 5.247.10^−2^ atomic unit that's indicated is automatically chosen to represent the most negative and then the most positive ESP on that surface of constant electron density. As a result, the MEP plot increases in the following way: Red < Orange < Yellow < Green < Blue [46, 47]. These sites give information about the region where the compound can have intermolecular interactions. We observe from Figure 5 that the mainly blueish color is localized on the hydrogen atoms of the methyl groups and the thiazole ring. Then, the reddish tinge is shown on both ends of the molecules near the oxygen (O) atoms. Both results make sense; oxygen atoms tend to be very electronegative and draw in electric charge so that they will have negative ESPs near them. Hydrogen atoms tend to donate an electric charge to the atoms to which that they are bonded, so they will be electron‐poor and have a positive ESP in the vicinity.

### 3.7 NLO Effects

We intend to assess the NLO properties of the crystal formed by our 2‐EPTh molecule in both static and dynamic states at a frequency of 532 nm. For this purpose, studying the dimer may be more suitable, as it can offer more relevant information on how different interactions impact the properties of the crystalline material.

We conducted a study on the optical properties of the organic compound under investigation, both in its single and dimeric forms. We measured crucial parameters such as second hyperpolarizability (γ), first hyperpolarizability (β), isotropic polarizability (α), and electric dipole moment (μ). This analysis involved using two calculation functions, B3LYP and CAM‐B3LYP, with the same 6‐311G(d, p) basis set. Additionally, we conducted simulations to understand how the molecule's nonlinear behavior is affected by the environment. These simulations were carried out under two different conditions: in a gaseous atmosphere and in the presence of a polar protic solvent, ethanol. The equations used in the study are presented in Table S2, and the results obtained from theoretical calculations are summarized in Table 4.

**TABLE 4:** Results of static and dynamic simulations of NLO properties for the 2‐EPTh monomer and dimer as a function of frequency (λ = 532 nm) (where α : 1 a.u. = 0.1482 × 10^−24^ esu and β : 1 a.u. = 8.63922 × 10^−33^ esu [48]).

		Monomer		Dimer	
		B3LYP		B3LYP	
		Gaz	Ethanol	Gaz	Ethanol
**Dipole moment**	**µ (D)**	1.81	2.75	0.76	0.95
**Polarizability Static**	**α (0,0) (u.a)**	324.40	410.51	705.96	891.12
	**α × 10** ^**−23**^ **(0,0) (esu)**	4.81	6.08	10.46	13.21
**Polarizability Dynamic**	**α (‐ω;ω) (u.a)**	351.98	383.97	765.67	829.03
	**α × 10** ^**−23**^ **(‐ω;ω) (esu)**	5.22	5.69	11.35	12.29
**1** ^**st**^ **static hyperpolarizability**	**β (0;0,0) (u.a)**	787.09	1450.70	284.89	504.53
	**β × 10** ^**−30**^ **(0;0,0) (esu)**	6.80	12.53	2.46	4.36
**1** ^**st**^ **dynamic hyperpolarizability**	**β (‐ω;ω,0) (u.a)**	1000.40	1382.38	326.17	434.39
	**β × 10** ^**−30**^ **(‐ω;ω,0) (esu)**	8.64	11.94	2.82	3.75
**2** ^**nd**^ **static hyperpolarizability**	**γ × 10** ^**−36**^ **(0;0,0,0) (esu)**	65.31	123.73	181.64	291.46
**2** ^**nd**^ **dynamic hyperpolarizability**	**γ × 10** ^**−36**^ **(‐ω;ω, 0,0) (esu)**	124.66	195.11	362.30	473.04

**FIGURE 6:**
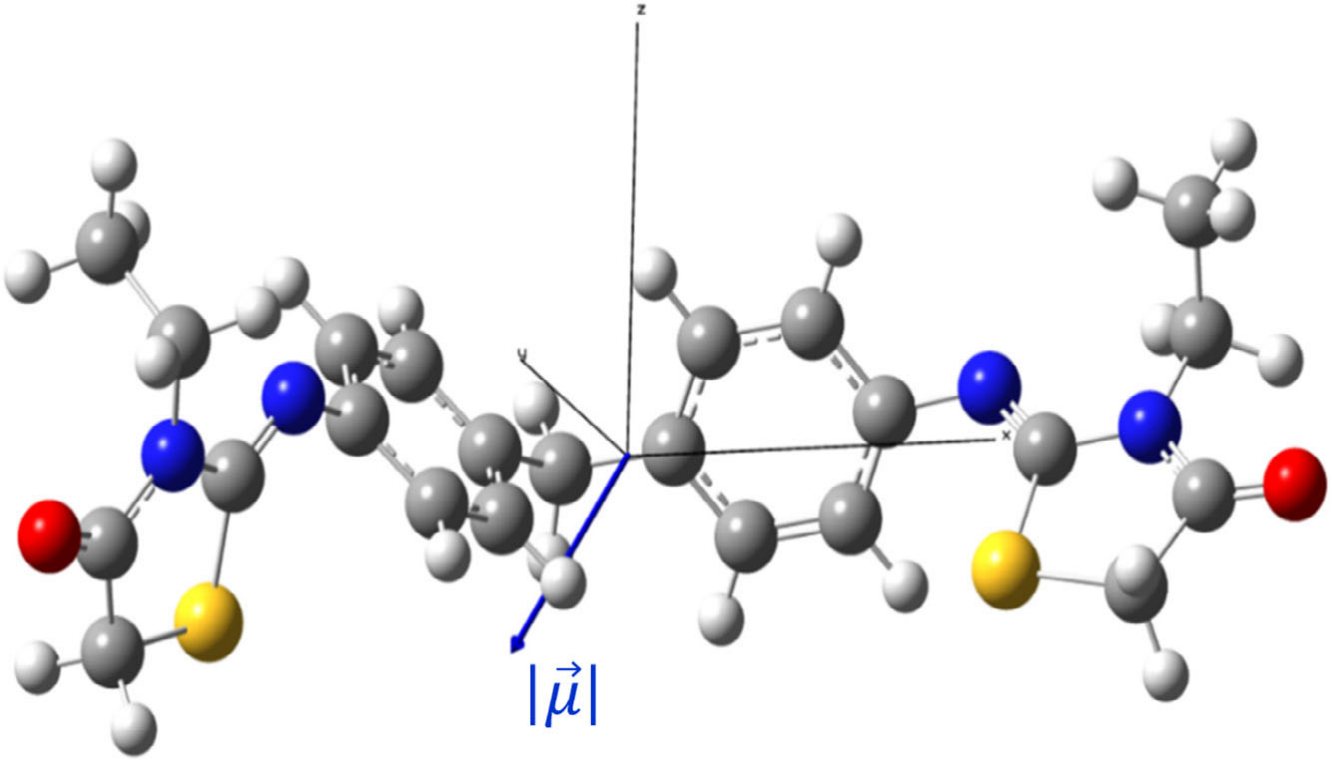
Orientation of the molecular dipole moment for the title compound.

The obtained results indicate a trend toward the cancelation of the dipole moment (μ) (Figure 6), which can be explained by the presence of symmetry in our molecule. The polarizability increased in the presence of a polar solvent compared to when the two functionalities, B3LYP and CAM‐B3LYP, were in a gaseous medium. Despite previously observing a low dipole moment, our monomer and dimer molecules can become polarized in a polar medium almost four and eight times better than pNA (α = 1.7 × 10^−23^ esu) [49], respectively.

Furthermore, we have also observed low values of the first hyperpolarizability (β), and no significant improvement is observed when adjusting the study parameters. This observation can be attributed to molecular symmetry, which affects the results of NLO by determining which electronic transitions are allowed and how the electron responds to light interaction. For example, in a centrosymmetric system, if the incident oscillating field at position “r” changes from plus “E” to minus “E,” it must also change from plus “E” to minus “E” at “‐r.” However, the polarizability in a second‐order system is proportional to “E^2^,” meaning it does not change sign. As a result, the first‐order optical phenomenon only occurs in noncentrosymmetric systems.

In both gaseous and polar solvent environments, there is a significant increase in second‐hyperpolarizability. This increase is observed at both static and dynamic levels. The formation of the molecular dimer is characterized by notably higher static hyperpolarizability values (181.64 × 10^−36^ esu in the gas phase and 291.46 × 10^−36^ esu in ethanol), almost three times higher than those obtained for the monomer in both environments (gas and polar solvent). Furthermore, compared to the reference pNA [49] (γ=15.5 × 10^−36^ esu), these values experience an increase of approximately 12 times in the gas phase and nearly 19 times in the presence of the solvent in the static state. Regarding dynamic hyperpolarizability, the dimer shows significantly increased values in the gas phase (362.30 × 10^−36^ esu), nearly three times higher than those of the monomer. In a polar medium, there is an improvement of about four times compared to the previously obtained results for the monomer.

### 3.8 Experimental Atomic and Molecular Properties

The title chemical's electron density and ESP have been analyzed using DFT and diffraction techniques. To start the electron density refinement with the most accurate positional and thermal parameters, MOPRO was used to do an initial high‐order spherical atom refinement on the complex structure's nonhydrogenated atoms. During the second refinement stage, the initial electron density parameters were obtained from the multipolar database, which contains descriptions of nonspherical atoms representing various chemical groups. The atomic and molecular properties, such as ESP, dipole moments, and interaction energies, may exhibit uncertainty due to the impact of restrictions [50, 51, 52].

#### 3.8.1 Dipole Moment

The experimental diffraction data, analyzed using the multipolar Hansen–Coppens model, yielded a total dipole moment of 4.66 D. The formula for determining molecular dipole moments relies on atomic net charges and atomic dipoles as the fundamental components, with the origin positioned at the center of mass.

$${µ}_{\mathrm{total}}=\sum_{i}{µ}_{i}+\sum r_{I}q_{i}$$

The experimental measurement of the total dipole moment exhibited a much greater magnitude than that calculated through theoretical computation. The experimental dipole moments in crystals, obtained by multipolar refinements of X‐ray data, were often seen to be greater than the ab initio estimated dipole moments for isolated molecules in the gas phase due to induced polarization. The findings align with previous research [53], indicating that the dipole moment was notably affected by the refining approach employed, such as the kappa, multipolar, and virtual‐atom models. Furthermore, even a minor adjustment in the handling of hydrogen atoms can significantly impact the computed magnitude and alignment of the dipole moment. The refinement process might result in an excessive number of parameters in the multipolar model, which, although it may improve the residuals, often leads to poorer dipole moments. However, the topological characteristics, such as position, density, and Laplacian at the crucial point, exhibited similarities.

### 3.9 Total ESP

**FIGURE 7:**
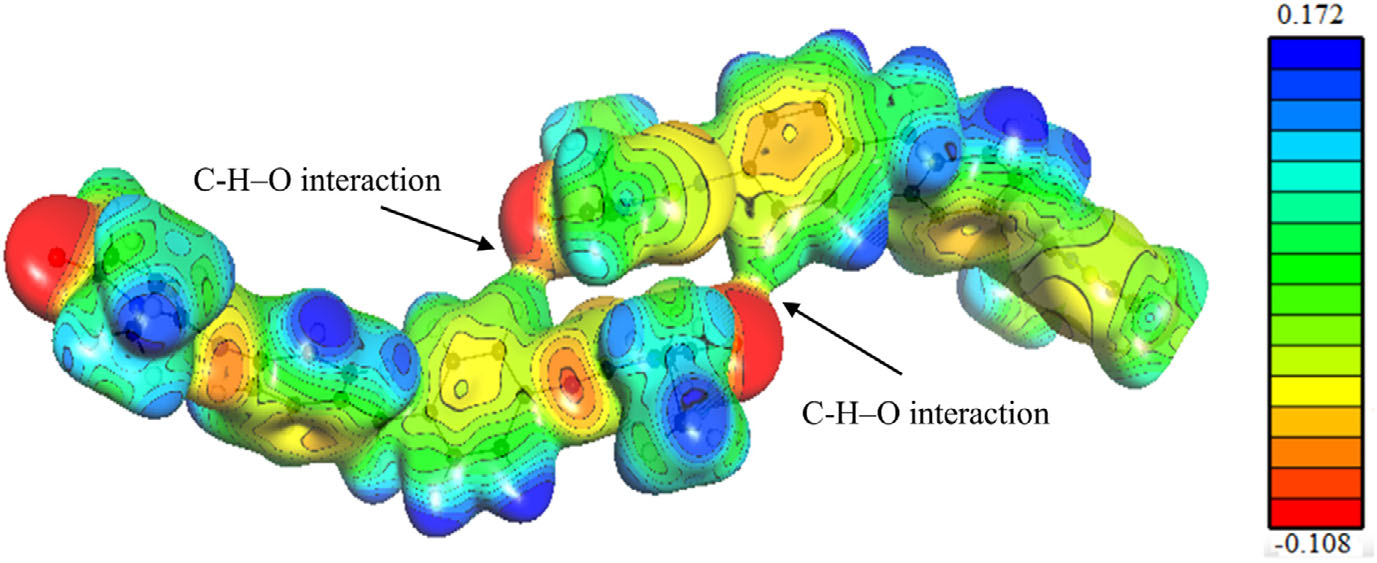
The electron density created by the 2‐EPTh dimer ‐X;‐Y;‐Z + 2 on the 0.01 eÅ^−3^ isosurface produces an ESP. The ESP exhibits its most extreme negative value (red) at −0.108 eÅ^−1^ and its most extreme positive value (blue) at 0.172 eÅ^−1^.

The MoPro Viewer program enables the computation of electrostatic parameters based on the charge‐density description acquired from the improved geometry of the system under study. This property is a crucial tool for predicting nucleophilic and electrophilic sites and comprehending intermolecular interactions. The ESP produced by the 2‐EPTh dimer was calculated, and the 0.01 eÅ^−3^ electron‐density surface was colored using a graduated color scheme based on the ESP value, as shown in Figure 7. The agreement between the previously mentioned DFT‐MEP and experimental maps is typically satisfactory since the positive and negative potential zones exhibit high similarity [54]. The ESP exhibits a maximum negative value (red) of −0.108 eÅ^−1^ and a maximum positive value (blue) of 0.172 eÅ^−1^. Consequently, the experimental potentials exceeded the ones estimated using DFT, possibly because of the polarization created within the crystal. Furthermore, the molecular surface's three‐dimensional ESP calculation highlights the specific binding locations of the 2‐EPTh dimer. The crystal's chemical group interactions are investigated by calculating the electrostatic energy utilizing the most recent improvements of the MoPro crystallographic software, as shown in Figure 7.

### 3.10 AIM Study

The AIM analysis was performed to investigate the intermolecular interactions and covalent bonding characteristics of the studied compound [55]. The electron density *ρ*_bcp_ (r), Laplacian ∇^2^ *ρ*(**r**), ellipticity (ϵ), and eigenvalues (*λ*_1_, *λ*_2_, *λ*_3_) [56] at the bond critical points (BCPs) were calculated using the MoPro software.

### 3.11 Topological Analysis of Covalent Bonds

The topological parameters of the covalent bonds are summarized in Table S3. The S—C bonds exhibited bond lengths of approximately (1.79 ± 0.02 Å) with electron density values around (1.17 ± 0.02 eÅ^−3^). The Laplacian values varied between positive and negative values depending on the bonding environment, indicating variations in electron concentration along the bond paths.

Aromatic C—C bonds showed higher electron densities (2.13 ± 0.03 eÅ^−3^) and strongly negative Laplacian values (−18.64 ± 0.68 eÅ^−5^), consistent with their π‐character. In contrast, nonaromatic C—C bonds displayed lower electron densities and less negative Laplacian values.

The C—O bonds presented the highest electron density (2.76 eÅ^−3^) and strongly negative Laplacian values (−20.54 ± 0.28 eÅ^−5^), confirming their strong covalent character. Similarly, the C—N bonds showed electron density values ranging from 1.68 to 2.22 eÅ^−3^ depending on the bonding environment.

### 3.12 Deformation and Laplacian Electron Density

**FIGURE 8:**
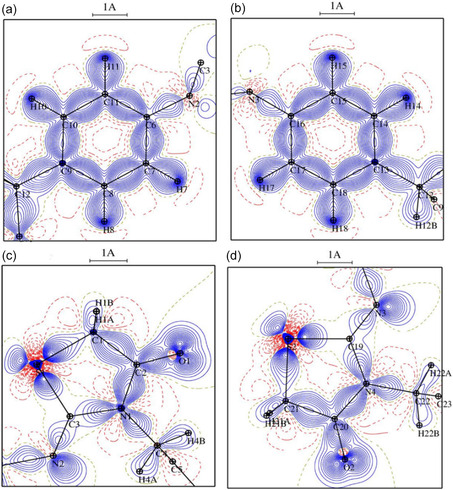
The positive (blue) and negative (red) contours represent the static deformation electron density distribution in the plane of the aromatic ring (a,b) and the thiazolidine ring (c,d). The intervals between each contour are 0.05 e Å^−3^.

**FIGURE 9:**
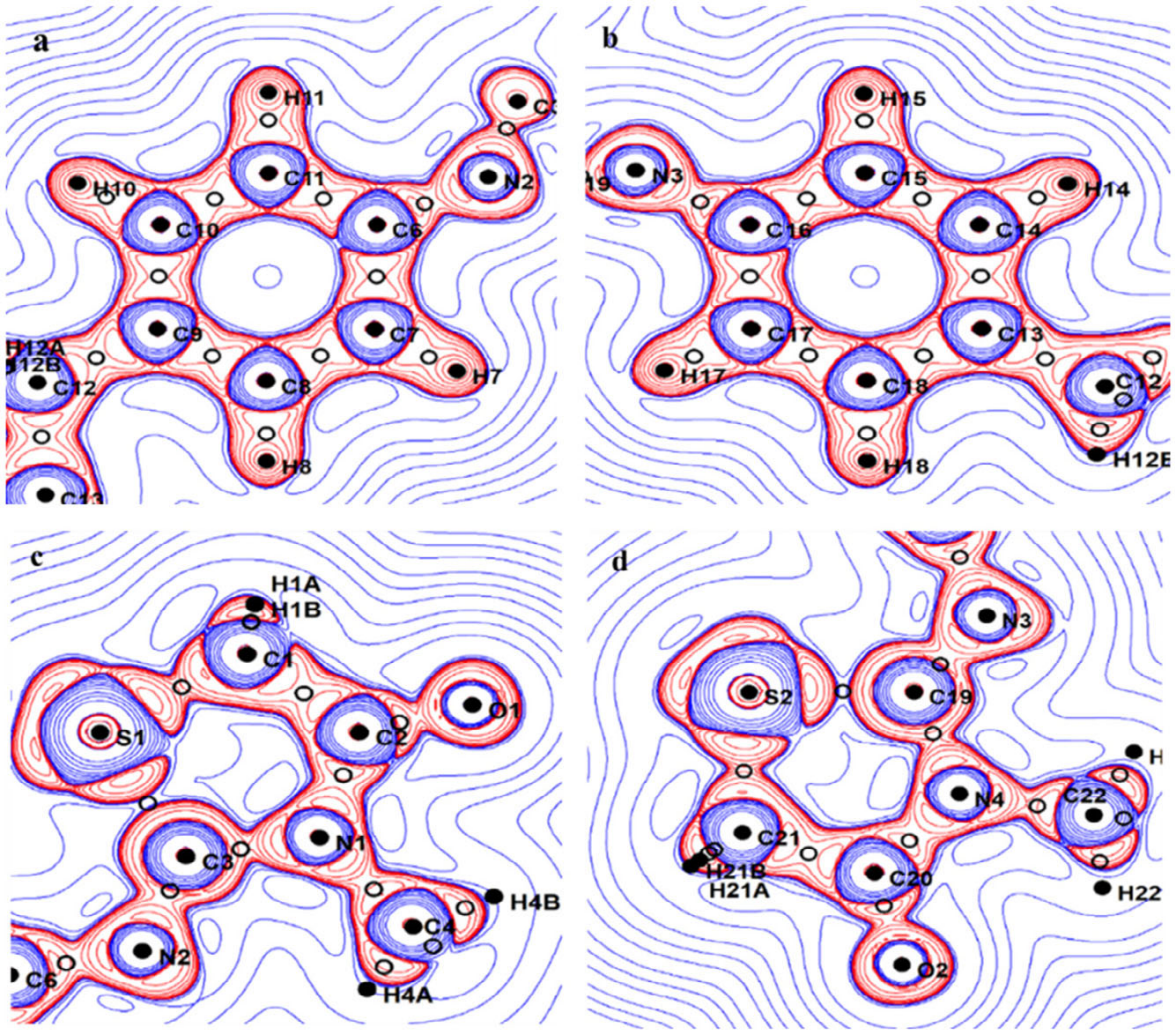
Laplacian representation of electron density in the plane of the thiazolidine ring (c,d) and the aromatic ring (a,b). ±2 e/Å^−5^ contour: Positive lines are blue, whereas negative lines are red.

The experimental deformation density maps (Figure 8a–d) visually illustrate the electron density distribution in the title compound, highlighting the bonding areas and electron lone pairs. Consistent with the topological findings, the static deformation density maps exhibit the following characteristics: There is a significant concentration of electron density in the C—S bond, namely toward the C atoms. The lack of regularity in the thiazolidine ring [57] is seen in the Laplacian maps shown in Figure 9c,d. As previously mentioned, we categorize the C—S bonds within the ring into two types. Both sulfur atoms exert an attractive force on electrons by shifting all associated BCPs toward the carbon atoms. The Laplacian at CP Figure 9c,d is contingent upon the nature of the atoms that are chemically bound to carbon. The atom C3 is double‐bound to N2 and has a single bond connecting it to N1. However, C1 is connected to C1 and hydrogen atoms (H1A, H1B). The electron concentration at S1–C1 and the depletion at S1–C3 in Figure 9c were observed. In addition, the unshared electron pair (LP) for the nitrogen, oxygen, and sulfur atoms was observable in the static deformation density maps.

### 3.13 Intramolecular Interactions

Van der Waals interactions, ionic bonds, and hydrogen bonds exhibit a (3, −1) critical point, which is characterized by a decrease in density and a positive Laplacian. This indicates the presence of feeble interatomic interactions.

**FIGURE 10:**
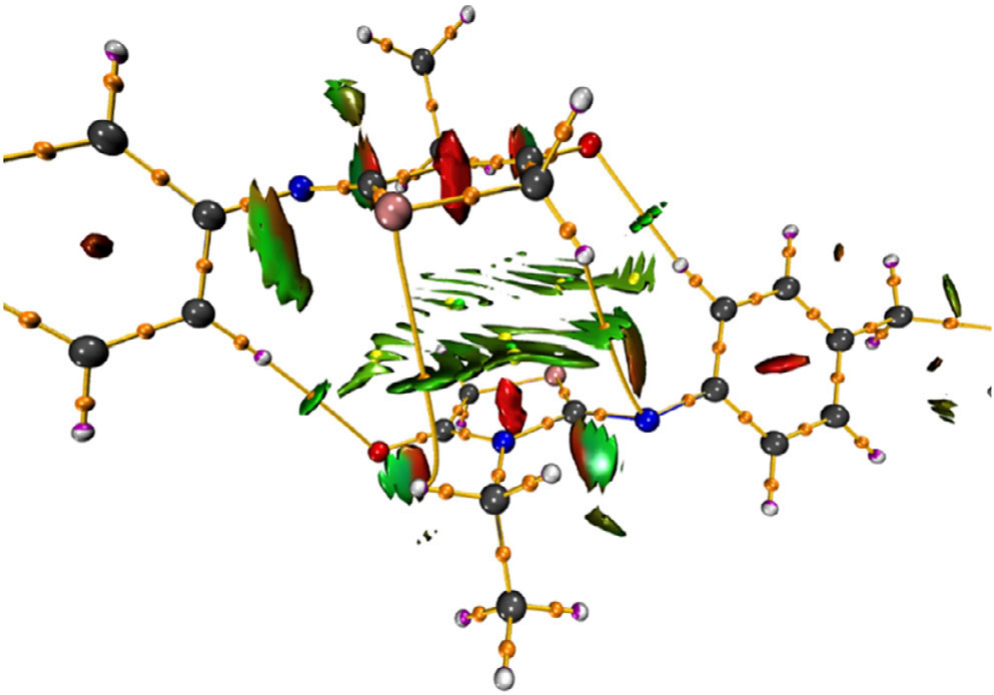
The three‐dimensional isosurface scatter plot depicts the intramolecular interactions of the title compound CP (3, +1) in yellow, CP (3, +3) in green, and CP (3,–1) in orange, with an isosurface distance of 0.6.

Abramov and Espinosa [58, 59], who extended on the proposals of Cremer and Kraka [60], propose that the local energy density attributes, which may be obtained from experimental data, can also be utilized to define chemical interactions using the approximation function. The covalent character is “proportional” to the potential energy V(cp), whereas the ionic character is “proportional” to the kinetic energy density G(cp). Once both energy densities are known, it is possible to distinguish between shared and closed–shell interactions [58, 59]. If the sum of G(cp) and V(cp) (total energy density), H(cp) is negative, the interactions are categorized as shared shells. The interaction is categorized as a closed shell because the kinetic energy density is dominant. The distance H17…O2 in our investigation (Table 5) was 2.30 Å, with an electron density at CP of 0.084 eÅ^−3^ and a Laplacian of 0.98 eÅ^−5^. The stabilizing energy of the hydrogen bond is 9 kJ/mol. Based on Koch and Popelier criteria [61, 62] and Guru Row and Munshi's conditions [63], these characteristics will lead to the same conclusion: this interaction is a weak to moderate hydrogen bond. The Mopro software calculates an experimental total electrostatic energy of 26.63 kJ/mol [64], classifying this interaction as a weak hydrogen interaction. Furthermore, weaker intramolecular interactions between S2…N4 and C19…H21 are reported in Table 5, with total electrostatic energies of 7.44 and 0.64 kJ/mol, respectively. Furthermore, bond critical sites (3, +1) known as ring critical points with ellipticity greater than one were well discovered (Figure 10), located at the center of the thiazolidine and aromatic rings. Furthermore, BCPs (3, +3) known as cage CPs were calculated with ellipticity < 0, positioned in the center between the two thiazolidine rings (Figure 10).

**TABLE 5:** Properties of intramolecular interactions with adjacent molecules through symmetry operations ‐X;‐Y;‐Z + 2.

A1	A2	D_12_ (Å)	G_cp_, kJ/mol	V_cp_, kJ/mol	ρ, eÅ^−3^	eÅ^−5^	λ_1_	λ_2_	λ_3_	ELLI.
S2	N4	3.74	6.57	−4.41	0.027	0.32	−0.05	−0.01	0.38	2.93
O2	H17	2.30	22.71	−18.86	0.084	0.98	−0.35	−0.35	1.67	0.01
C19	H21	3.07	7.69	−4.93	0.026	0.38	−0.04	−0.02	0.45	0.77

## 4 Conclusion

The (Z)4,4^′^‐bis[‐3‐N‐ethyl‐2‐N’‐(phenylimino) thiazolidin‐4‐one] methane composite was studied further using theoretical and experimental spectroscopic techniques such as 1H and 13C NMR, vibrational frequencies (IR), and ultraviolet–visible spectroscopy (UV–vis). These experimental results agree with those of molecular modeling and confirm the structural analysis and synthesis of the title molecule. We analyzed the molecule's electrical characteristics, estimated and identified any charge transfer interactions using the B3LYP function with the 6‐311G (d, p) basis set. A theoretically and experimentally derived MEP map of the molecule demonstrates that hydrogen atoms represent positive likely regions and oxygen atoms represent negative likely regions. These findings support the existence of intermolecular interactions. The detected energy gap value can be used to deduce the chemical reactivity and kinetic stability of the compound under investigation, as well as identify any charge transfer interactions that may be occurring within the molecule. Furthermore, the DOS spectra illustrate the molecular orbital compositions and their contributions to chemical bonding. In addition, utilizing the AIM approach to investigate numerous intermolecular interactions, we discovered the presence of multiple weak to moderate hydrogen bonds. The Mopro software also computed an experimental total electrostatic energy of 26.63 kJ/mol, validating the connection as a weak hydrogen bond. Finally, estimating NLO properties revealed that 2‐EPTh could be of interest as a molecule for NLO applications. A comparison investigation with different reference chemicals corroborated this. More than that, we obtained high values of gamma (γ). These high gamma values indicate an even stronger optical response than beta (β) values, as gamma is related to higher‐order optical effects. Additionally, the presence of solvent, specifically polar solvents, enhanced the NLO properties of our molecule. Interactions between solvent molecules and those of the material can modify the electronic and structural properties of the material, leading to an increase in the efficiency of nonlinear optical processes.

## Supporting Information

Additional supporting information can be found online in the supporting information section.

## Author Contributions

**Zohra Douaa Benyahlou**: methodology, investigation, writing – original draft preparation. **Fayssal Triki Baara**: investigation, data curation. **Salem Yahiaoui**: methodology, software, formal analysis, investigation. **Merzouk Saidj**: software: investigation. **Keltoum Dermeche**: investigation. **Mohammed Harir**: investigation. **Youcef Megrouss**: formal analysis, visualization. **Ahmed Djafri**: formal analysis, writing – review and editing. **Abdelkader Chouaih**: Conceptualization, validation, resources, writing – review and editing. **Hamdi Bendif**: writing – review and editing, supervision, project administration, funding acquisition. **Walid Elfalleh**: project administration. **Tarek H. Taha**: supervision. **Anis Ahmad Chaudhary**: funding acquisition. **Stefania Garzoli**: supervision. All authors have read and agreed to the published version of the manuscript.

## Funding

This work was supported and funded by the Deanship of Scientific Research at Imam Mohammad Ibn Saud Islamic University (IMSIU) (grant number IMSIU‐DDRSP2602).

## Conflicts of Interest

The authors declare no conflicts of interest.

## Supporting information

Supplementary Material

## Data Availability

No data was used for the research described in the article.
