# Supplementary material for: Atom‐in‐Molecule Analysis, Density Function Theory Research, Spectroscopy, Charge Transfer, and (Static, Dynamic) Nonlinear Optical Effects of (Z)4,4′‐Bis[‐3‐N‐Ethyl‐2‐N’‐(Phenylimino) Thiazolidin‐4‐One] Methane
Source: ChemistryOpen. 2026 May 21;15(6):e70205. doi: 10.1002/open.70205 (PMC13240089; doi:10.1002/open.70205)
Supplement: Supplementary file 1 — Supplementary Material [file OPEN-15-e70205-s001.pdf]

AIM Analysis, DFT Research, Spectroscopy, Charge transfer, and (static, dynamic) NLO Effects of (Z)4,4'-bis[-3-N-ethyl-2-N'-(phenylimino) thiazolidin-4-one] methane

Zohra Douaa Benyahlou[a], Fayssal Triki Baara[b], Salem Yahiaoui[a], Merzouk Saidj[a], Keltoum Dermeche[b,c], Mohammed Harir[c,d], Youcef Megrouss[a,e], Ahmed Djafri[a,f], Abdelkader Chouaih[a], Hamdi Bendif[g]*, Elfalleh Walid[g], Tarek H. Taha[g], Anis Ahmad Chaudhary[g], Stefania Garzoli[h]*

[a]*Process Engineering Department, Laboratory of Technology and Solid Properties, Faculty of Sciences and Technology, Abdelhamid Ibn Badis University, Mostaganem, Algeria; benyahlou.zohra.douaa@gmail.com; sifislem2010@gmail.com; saidj.merzouk@yahoo.de; youcefmegrouss@gmail.com; djafahmed@yahoo.fr; achouaih@gmail.com;*
[b]*Laboratory of Environment and Sustainable Development, Faculty of Nature and Life Sciences, University of Ahmed Zabana, Bourmadia, 48000, Relizane, Algeria. bn.zohra.douaa@gmail.com; keltoum.dermeche@univ-usto.dz;*
[c]*Faculty of Nature and Life Sciences, University of Sciences and Technology of Oran-Mohamed Boudiaf, Oran 31000, USTO-MB, B.P. 1505, El-Mn'aour, Oran 31000, Algeria. Mohammed.harir@univ-usto.dz*
[d]*Applied Organic Synthesis Laboratory, Faculty of Exact and Applied Sciences, Department of Chemistry, University Oran-1, Algeria*
[e]*Chemistry Department, Faculty of Exact Sciences and Informatic, Hassiba Benbouali University, Chlef, Algeria*
[f]*Organic Synthesis Division, Centre de Recherche Scientifique et Technique en Analyses Physico-Chimiques (CRAPC), Tipaza, Algeria.*
[g]*Biology Department, College of Science, Imam Mohammad Ibn Saud Islamic University (IMSIU), Riyadh, 11623, Saudi Arabia; hlbendif@imamu.edu.sa; wbelfallah@imamu.edu.sa; thali@imamu.edu.sa; aachaudhary@imamu.edu.sa;*
[h]*Department of Chemistry and Technologies of Drug, Sapienza University, 00185 Rome, Italy; stefania.garzoli@uniroma1.it*

***Correspondence:** hlbendif@imamu.edu.sa; stefania.garzoli@uniroma1.it,

Table S1. Vibrational wavenumbers obtained for 2-EPTh compound at B3LYP/6-311G(d,p) basis set.

No.	Experimental	B3LYP /6-311G(d, p)		Relative intensity	Assignments with PED>10
		Unscaled	Scaled		
159		3187	3101	6.1545	υCH (91) ring2
158		3186	3100	3.9085	υCH (86) ring3
157		3184	3098	13.2819	υCH_{asy} (26) ring3+ υCH (72) ring3
156	3099	3184	3098	11.3649	υCH (83) ring2
155		3164	3079	11.9849	υCH (84) ring2+ υCH_{asy} (16) ring2
154		3162	3077	12.5306	υCH_{asy} (15) ring3+ υCH (85) ring3
153	3072	3156	3071	16.6225	υCH (91) ring3
152		3155	3070	17.9358	υCH (91) ring2
151		3138	3053	8.2794	υCH (52) + υCH_{asy} (34)

150		3138	3053	11.4681	υCH (53) + υCH_{asy} (34)
149		3127	3043	0.3111	υCH_{asy} (48) ring4+ υCH (52) ring4
148		3127	3043	0.3250	υCH_{asy} (48) ring1+ υCH (51) ring1
147		3113	3029	13.9748	υCH_{asy} (28) + υCH (60)
146		3112	3028	14.6031	υCH_{asy} (26) + υCH (60)
145		3104	3020	27.8952	υCH_{asy} (44) + υCH (44)
144		3104	3020	25.7705	υCH_{asy} (54) + υCH (45)
143		3079	2996	8.1400	υCH (83)
142		3079	2996	9.0465	υCH (83)
141		3079	2996	10.8271	υCH_{asy} (91)ring4
140		3078	2995	10.2335	υCH_{asy} (91)ring1
139	2976	3050	2967	12.1660	υCH_{asy} (45) + υCH (55)
138	2957	3038	2956	28.2130	υCH (81)
137		3038	2956	12.5644	υCH_{asy} (81)
136		3018	2937	33.9520	υCH (100)
135		1798	1750	157.8920	υCO (76) ring4
134	1721	1798	1749	228.1375	υCO (76) ring1
133		1709	1663	698.5503	υNC (73) ring1,4
132		1704	1658	1642.3830	υNC_{asy} (33) ring1,4+ υNC (40) ring1,4
131	1627	1649	1604	67.8827	υCC (27) ring2,3
130		1645	1601	130.1189	υCC (14) ring2,3+ υCC_{asy} (13) ring2,3
129		1605	1562	6.0289	υCC (27) ring2,3 + υCC_{asy} (24) ring2,3
128		1600	1556	4.8051	υCC_{asy} (26) ring2,3 + υCC (27) ring2,3
127	1504	1538	1496	19.3032	σHCC (10) ring2,3
126		1535	1494	123.2732	σHCC_{asy} (10) ring2,3
125		1505	1464	4.3744	σHCH_{asy} (19) + σHCH (53) + τ$HCCN_{asy}$ (12)
124		1505	1464	3.5297	σHCH_{asy} (19) + σHCH (53) + τ$HCCN_{asy}$ (12)
123		1496	1456	9.7339	σHCH (45) + σHCH_{asy} (30) + τHCCN (15)
122		1496	1455	9.9027	σHCH (46) + σHCH_{asy} (30) + τHCCN (15)
121		1483	1443	6.7666	σHCH (83)
120		1475	1435	29.1285	σHCH (76)
119	1433	1473	1433	32.5960	σHCH (78)
118		1462	1423	9.2362	σHCH (89) ring4
117		1461	1422	10.6276	σHCH (89) ring1
116		1445	1406	4.3113	
115		1443	1404	1.3089	υCH (12) ring2,3 + υCH_{asy} (11) ring2,3
114		1418	1380	18.5507	σHCH_{asy} (40) + τHCNC (19)
113		1418	1379	25.8946	σHCH_{asy} (39) + τHCNC (19)
112	1366	1404	1366	121.4341	υNC (18) ring1 + σHCC (28) ring1 + τ$HCNC_{asy}$(10) ring1
111	1366	1404	1366	97.8276	υNC (18) ring4 + σHCC (28) ring4
110		1400	1362	7.8266	σHCH (34) + τ$HCNC_{asy}$ (11) + τHCNC (26)
109		1399	1362	7.4289	σHCC (10) + σHCH (22) + τ$HCNC_{asy}$ (11) + τHCNC (29)
108		1358	1321	261.5059	
107		1357	1320	74.2608	
106		1355	1319	129.6315	τHCCC (18)
105	1341	1347	1310	6.0895	σHCC_{asy} (10) ring2,3 + σHCC (16) ring2,3
104		1320	1284	5.6824	σHCC (11) ring2,3
103		1314	1279	0.3959	υCC (32) ring2,3
102		1295	1260	14.8942	
101		1293	1258	10.7246	υNC (12) ring1,2 + σHCC_{asy} (15) ring1,2
100	1256	1291	1256	4.9916	σHCC_{asy} (10) ring3,4
99		1253	1219	28.7453	τ$HCSC_{asy}$(38) ring4 + τHCSC(44) ring4
98		1253	1219	22.6406	τ$HCSC_{asy}$(37) ring1 + τHCSC(44) ring1

97		1225	1192	50.8818	
96	1198	1224	1191	75.1240	υCC$_{asy}$ (14) + υCC (13)
95		1222	1189	120.4968	υCC (10)
94		1209	1176	4.3971	υCC (29)
93		1199	1167	0.3385	σHCC (37) + τHCCC (32)
92		1195	1163	7.8548	σHCC (26) + σHCC$_{asy}$ (10)
91		1194	1162	44.8450	σHCC (38)
90	1121	1149	1118	1.6225	σHCS (50) ring4+ τHCSC$_{asy}$(27) ring4
89		1148	1117	1.7506	σHCS (50) ring1 + τHCSC$_{asy}$(27) ring1
88		1132	1102	61.9604	υCC$_{asy}$ (10) + τHCCN$_{asy}$ (10)
87		1132	1101	33.4449	
86		1131	1101	159.6881	υCC$_{asy}$ (13) + τHCCN$_{asy}$ (13)
85		1125	1095	5.4200	σHCC$_{asy}$ (12)
84		1109	1079	11.0773	υCC (15) + σHCH (11) + τHCCN$_{asy}$ (15) ring1+ τHCCN (29) ring1
83		1109	1079	13.3549	υCC (15) + σHCH (11) + τHCCN$_{asy}$ (15) ring4+ τHCCN (29) ring4
82	1046	1053	1025	13.6932	υCC (41) + υNC$_{asy}$ (26) ring4
81		1052	1024	16.1113	υCC (42) + υNC$_{asy}$ (26) ring1
80	1010	1033	1005	11.8067	σCCC$_{asy}$ (68) + σCCC (17)
79		1033	1005	5.1864	σHCC (10) + σCCC (60) + σCCC$_{asy}$ (14)
78		974	948	0.0277	τHCCC$_{asy}$ (38) ring2 + τHCCC (32) ring2 +τCCCC$_{asy}$ (13) ring2
77		971	945	0.0276	τHCCC$_{asy}$ (26) ring3 + τHCCC (32) ring3
76		964	938	2.2140	υCC (11) + υCC$_{asy}$ (14)
75		964	937	5.6375	υCC (16) + υCC$_{asy}$ (23)
74		962	936	1.0108	υCC (11) + τHCCC (11)
73		959	933	0.5013	τHCCC (20) + τHCCC$_{asy}$ (15) + τCCCC$_{asy}$
72		930	905	6.5453	(18)
71	900	922	897	7.5401	σHCC$_{asy}$ (15) + τHCCC (17)
70		921	896	7.0690	σHCS$_{asy}$ (47) ring4 + τHCSC$_{asy}$(19) ring4 + γOCNC (13) ring4
69		904	879	30.6618	σHCS$_{asy}$ (47) ring1 + τHCSC$_{asy}$(19) ring1+ γOCNC (13) ring1
68		902	877	57.1019	
67		871	847	17.1754	
66		855	832	1.0435	
65		846	823	16.4776	υCC (21)
64		840	817	3.3810	τHCCC (14)
63		838	816	1.6191	τHCCC$_{asy}$ (16) + τHCCC (50)
62		835	812	18.1034	τHCCC (52) + τHCCC$_{asy}$ (10)
61	777	803	781	3.8724	τHCCC (31)
60		793	772	16.3101	υCC$_{asy}$ (10)
59		790	768	1.2821	
58		789	768	3.9519	σCCN (14)
57	759	766	745	11.0596	σCCN (10)
56		761	741	34.2569	σCCN (13)
55		735	715	2.6040	σCCN$_{asy}$ (11)
54		727	708	2.0252	τCCCC$_{asy}$ (25)
53		663	645	6.7273	
52		662	644	3.6755	σCCC$_{asy}$ (20) + σCCC (16) + σNCC (10)
51		638	621	15.3173	σCCC (14) + σCCC$_{asy}$ (22) + σNCC (10)
50		631	614	9.2231	
49		628	611	8.4586	
48		624	607	5.3074	σCNC$_{asy}$ (11)

47		614	597	13.7904	υSC (81) ring1 + σCNC (15)
46		611	595	7.7056	γSNNC (47)
45		595	579	1.4728	γ$SNNC_{asy}$ (21) + γSNNC (27)
44		587	571	2.2818	
43		586	570	6.0019	γOCNC (47)
42		564	549	3.6833	γOCNC (26) + γ$OCNC_{asy}$ (16)
41	526	522	508	15.2145	υNC (10)
40		510	496	14.0502	γ$NCCC_{asy}$ (13) + γ$CCCC_{asy}$ (16)
39		502	488	20.1447	σNCC_{asy} (15) + τNCNC (10) + γ$NCCC_{asy}$ (10)
38		496	482	17.5002	υSC_{asy} (16)+ σSCN (13) + σCCN (11)
37		473	460	11.7877	υSC_{asy} (25)+ σNCN (11)
36		467	454	9.6357	σSCN (11) + σOCC_{asy} (13)
35		446	434	3.8024	σSCN_{asy} (12) + σOCC (17) + σCNC (11)
34		434	423	10.5093	σNCC (20) + τNCNC (10)
33		419	407	1.2539	σNCC (13) + γCCCC (12)
32		416	405	1.1053	γCCCC (41)
					γCCCC (42)

Ring1(C21 C20 N4 C19 S2) , Ring2(C16 C15 C14 C13 C18 C17) , Ring3(C6 C7 C8 C9 C10 C11) , Ring4(C1 C2 N1 C3 S1), γ : Out of plane bending (OUT), β : In plane bending (BEND), υ : Stretching (elongation) (STRE), τ : twisting (TORS), σ : bending (BEND), ρ: rocking.

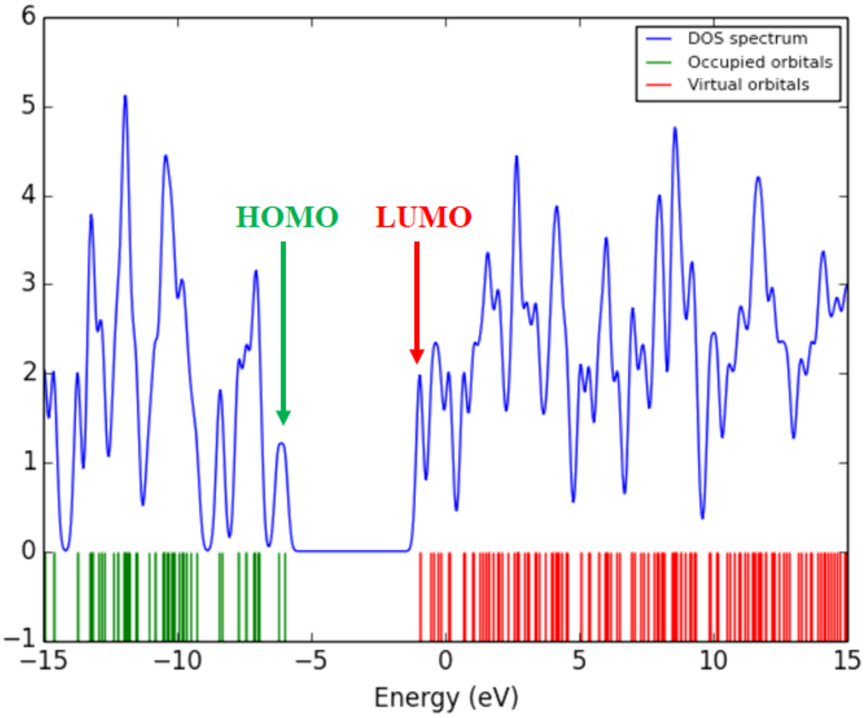

Fig. S1. DOS spectrum of 2-EPTh was obtained based on the B3LYP/6-311G(d,p) level.

Table S2. Equations used to calculate nonlinear optics parameters.

NLO properties	Equations
**Polarizability (α)**	$\alpha = \frac{1}{3}(\alpha_{xx} + \alpha_{yy} + \alpha_{zz})$
**First-order hyperpolarizability (β)**	$\beta_{tot} = (\beta_x^2 + \beta_y^2 + \beta_z^2)^{1/2}$<br>Where<br>$\beta_x = \beta_{xxx} + \beta_{xyy} + \beta_{xzz}$<br>$\beta_y = \beta_{yyy} + \beta_{yzz} + \beta_{yxx}$<br>$\beta_z = \beta_{zzz} + \beta_{zxx} + \beta_{zyy}$
**Second-order hyperpolarizability (γ)**	$< \gamma >= \frac{1}{5}[\gamma_{xxx} + \gamma_{yyy} + \gamma_{zzz} + 2(\gamma_{xxyy} + \gamma_{xxzz} + \gamma_{yyzz})]$
**Dipole moment (μ)**	$\mu = \sqrt{\mu_x^2 + \mu_y^2 + \mu_z^2}$

Table S3. Characteristics of the title compound bond critical point (3,-1).

A1	A2	D_{12}(Å)	D_{1cp}(Å)	D_{2cp}(Å)	ρ (eÅ^{-3})	∇^2 (eÅ^{-5})	λ_1	λ_2	λ_3	ELLI.
S2	C19	1.77	1.00	0.76	1.15	1.24	-5.76	-5.47	12.46	0.05
S2	C21	1.80	1.01	0.79	1.18	-1.36	-6.86	-6.27	11.77	0.09
S1	C3	1.77	1.01	0.77	1.14	1.33	-5.72	-5.39	12.43	0.06
S1	C1	1.80	1.01	0.79	1.19	-1.47	-6.95	-6.33	11.80	0.10
N3	C19	1.26	0.80	0.45	2.16	-5.48	-11.60	-11.53	17.65	0.01
N3	C16	1.41	0.80	0.61	1.86	-11.43	-13.47	-12.28	14.33	0.10
O1	C2	1.21	0.78	0.43	2.76	-20.82	-24.13	-22.65	25.96	0.07
N1	C3	1.40	0.83	0.57	1.83	-6.75	-10.65	-9.68	13.59	0.10
N1	C2	1.36	0.81	0.55	2.22	-20.66	-17.33	-14.92	11.60	0.16
N1	C4	1.47	0.85	0.62	1.68	-8.69	-11.28	-10.61	13.20	0.06
O2	C20	1.21	0.78	0.43	2.77	-20.26	-24.27	-22.81	26.82	0.06
N2	C6	1.40	0.80	0.60	1.87	-11.66	-13.61	-12.27	14.22	0.11
N2	C3	1.25	0.80	0.45	2.17	-4.93	-11.70	-11.62	18.39	0.01
N4	C19	1.39	0.83	0.57	1.85	-7.10	-10.74	-9.75	13.39	0.10
N4	C20	1.37	0.81	0.56	2.20	-19.65	-17.09	-14.68	12.12	0.16
N4	C22	1.46	0.85	0.62	1.68	-8.74	-11.41	-10.50	13.18	0.09
C15	H15	1.06	0.70	0.37	1.92	-21.31	-18.71	-17.71	15.11	0.06
C15	C14	1.39	0.70	0.70	2.14	-19.08	-16.23	-13.43	10.58	0.21
C15	C16	1.39	0.70	0.69	2.10	-18.14	-16.06	-12.84	10.76	0.25
C10	H10	1.08	0.71	0.37	1.86	-19.82	-17.94	-17.02	15.14	0.05
C10	C9	1.39	0.69	0.70	2.14	-18.58	-16.22	-13.39	11.03	0.21
C10	C11	1.39	0.69	0.69	2.15	-19.25	-16.29	-13.48	10.52	0.21
C6	C7	1.40	0.69	0.70	2.09	-17.89	-15.95	-12.76	10.83	0.25
C6	C11	1.40	0.69	0.70	2.10	-18.04	-16.02	-12.80	10.78	0.25
C12	H12A	1.09	0.69	0.40	1.82	-17.52	-15.99	-15.78	14.25	0.01
C12	H12B	1.08	0.69	0.40	1.83	-17.68	-15.96	-15.96	14.24	0.00
C12	C9	1.51	0.73	0.78	1.69	-10.32	-11.31	-10.42	11.40	0.09
C12	C13	1.50	0.73	0.78	1.71	-10.67	-11.27	-10.76	11.37	0.05
C14	H14	1.08	0.71	0.37	1.86	-19.88	-17.99	-17.02	15.13	0.06
C14	C13	1.39	0.69	0.70	2.16	-18.88	-16.34	-13.49	10.95	0.21

C16	C17	1.39	0.69	0.70	2.11	-18.30	-16.12	-12.89	10.71	0.25
C9	C8	1.40	0.70	0.69	2.14	-18.50	-16.19	-13.37	11.05	0.21
C8	H8	1.07	0.70	0.37	1.89	-20.56	-18.27	-17.42	15.13	0.05
C8	C7	1.39	0.69	0.69	2.14	-19.18	-16.26	-13.46	10.55	0.21
C2	C1	1.51	0.75	0.76	1.69	-10.48	-11.41	-10.60	11.52	0.08
C7	H7	1.07	0.70	0.37	1.90	-20.77	-18.42	-17.47	15.12	0.05
C17	H17	1.06	0.70	0.36	1.92	-21.35	-18.73	-17.72	15.11	0.06
C17	C18	1.39	0.70	0.70	2.13	-18.93	-16.17	-13.38	10.63	0.21
C20	C21	1.51	0.75	0.76	1.70	-10.56	-11.46	-10.61	11.51	0.08
C13	C18	1.39	0.70	0.69	2.16	-18.87	-16.33	-13.49	10.95	0.21
C18	H18	1.07	0.70	0.37	1.89	-20.48	-18.30	-17.32	15.14	0.06
C21	H21A	1.08	0.70	0.39	1.87	-18.31	-16.84	-16.66	15.19	0.01
C21	H21B	1.08	0.69	0.39	1.87	-18.34	-16.89	-16.64	15.19	0.01
C11	H11	1.08	0.71	0.37	1.87	-19.98	-17.98	-17.12	15.12	0.05
C4	H4A	1.07	0.69	0.39	1.91	-19.91	-17.68	-17.14	14.91	0.03
C4	H4B	1.08	0.69	0.39	1.91	-19.87	-17.65	-17.14	14.91	0.03
C4	C5	1.50	0.77	0.74	1.65	-9.58	-10.56	-10.15	11.13	0.04
C1	H1A	1.08	0.69	0.39	1.87	-18.50	-16.99	-16.69	15.18	0.02
C1	H1B	1.08	0.70	0.39	1.86	-18.18	-16.82	-16.55	15.19	0.02
C22	H22A	1.07	0.69	0.39	1.91	-19.94	-17.53	-17.30	14.89	0.01
C22	H22B	1.08	0.69	0.39	1.91	-19.90	-17.61	-17.19	14.89	0.02
C22	C23	1.51	0.77	0.74	1.64	-9.46	-10.47	-10.12	11.13	0.03
C23	H23A	1.07	0.67	0.39	1.81	-16.89	-15.89	-15.22	14.21	0.04
C23	H23B	1.07	0.68	0.39	1.81	-16.85	-15.86	-15.21	14.22	0.04
C23	H23C	1.07	0.68	0.39	1.81	-16.75	-15.80	-15.18	14.23	0.04
C5	H5A	1.07	0.68	0.40	1.80	-16.65	-15.69	-15.19	14.23	0.03
C5	H5B	1.07	0.68	0.40	1.80	-16.60	-15.61	-15.22	14.23	0.03
C5	H5C	1.07	0.67	0.39	1.81	-16.79	-15.73	-15.28	14.21	0.03

D_{12}: distance between two atoms, D_{1cp}, D_{2cp}: distance from the first and the second atom to the critical point, Laplacian [∇^2 ρbcp (eÅ^{-5})], electron density [ρbcp (eÅ^{-3})].
